# Global dominance of non-institutional delivery and the risky impact on maternal mortality spike in 25 Sub-Saharan African Countries

**DOI:** 10.1186/s41256-025-00409-x

**Published:** 2025-02-27

**Authors:** Oyewole K. Oyedele, Temitayo V. Lawal

**Affiliations:** 1 International Research Center of Excellence, Institute of Human Virology Nigeria, Abuja, Nigeria; 2 Department of Epidemiology and Medical Statistics, College of Medicine, University of Ibadan, Ibadan, Nigeria

**Keywords:** Non-institutional Delivery, Maternal Mortality, Global health, Lifetime Risk, Correlation, Regression, Sub-Saharan Africa

## Abstract

**Background:**

Despite 70% of global maternal death occurring in Sub-Saharan Africa (SSA) and the high rate of non-institutional delivery (NID), studies that inspect the connections are needed but lacking. Thus, we investigated the urban–rural burden and risk factors of NID and the correlate with maternal mortality to extend strategies for sinking the mortality spike towards sustainable development goal (SDG-3.1) in SSA.

**Methods:**

Secondary analysis of recent (2014–2021) cross-sectional demographic-health-survey (DHS) were conducted across 25-countries in SSA. Primary outcome was institutional versus non-institutional delivery and secondary outcome was maternal-mortality-ratio (MMR) per 100,000 livebirths and the lifetime risk (LTR), while predictors were grouped by socio-economic, obstetrics and country-level factors. Data were weighted to adjust for heterogeneity and descriptive analysis was performed. Pearson chi-square, correlation, and simple linear regression anlyses were performed to assess relationships. Multivariable logistic regression further evaluated the predictor likelihood and significance at alpha = 5% (95% confidence-interval ‘CI’).

**Results:**

Prevalence of NID was highest in Chad (78.6%), Madagascar (60.6%), then Nigeria (60.4%) and Angola (54.3%), with rural SSA dominating NID rate by about 85%. Odds of NID were significantly lower by 60% and 98% among women who had at least four antenatal care (ANC) visits (aOR = 0.40, 95%CI = 0.38–0.41) and utilized skilled birth attendants (SBA) at delivery (aOR = 0.02, 95%CI = 0.01–0.02), respectively. The odds of NID reduces by women age, educational-level, and wealth-quintiles. Positive and significant linear relationship exist between NID and MMR (ρ = 0.5453), and NID and LTR (ρ = 0.6136). Consequently, 1% increase in NID will lead to about 248/100000 and 8.2/1000 increase in MMR and LTR in SSA respectively.

**Conclusions:**

Only South Africa, Rwanda and Malawi had achieved the WHO 90% coverage for healthcare delivery. ANC and SBA use reduced NID likelihood but, MMR is significantly influenced by NID. Hence, strategic decline in NID will proportionately influence the sinking of MMR spike to attain SDG-3.1 in SSA.

**Supplementary Information:**

The online version contains supplementary material available at 10.1186/s41256-025-00409-x.

## Introduction

Global population of pregnant women in the lower-middle income countries (LMICs) continue to utilize the unsafe non-institutional delivery (NID) as the preferred mode of childbirth [1]. This is despite the high rate of pregnancy and childbirth related death which occurred every 2 min, leading to approximately 800 women deaths every day in 2020 [2, 3]. Meanwhile, about 95% of the global maternal mortality occurred in the LMIC with Sub-Saharan Africa (SSA) accounting for more than two-third [3, 4].

Thus, the global burden of maternal and newborn mortality hitherto resides in SSA as the recent maternal mortality ratio (MMR) revealed that South Sudan, Chad, and Nigeria with MMR of 1223, 1063 and 1047 per 100,000 livebirths in 2017–2020 respectively, have been consistent in the top 3 most affected countries in the world [2, 3, 5]. Although, the sustainable development goal (SDG) aims to reduce MMR to approximately 70 per 100,000 livebirths by 2030 [6], SSA countries will require a significant and rapid reduction to meet this target. Achieving this goal remain a formidable challenge as the global community approaches the 2030 deadline for SDG-3 which focuses on ensuring good health and wellbeing for all ages [6].

The prevalence of home births which does not guarantee safety of mother and newborn compared to the facility-based delivery is reportedly 56.8% (46.2% after pregnancy care uptake) in Nigeria [7, 8], 81% in Sudan [9], and 78% in Chad and about 30% in LMICs [10]. Furthermore, Poor pregnancy, labor and delivery care that includes non-utilization of antenatal care (ANC), the unsafe utilization of NID, absence of skilled birth attendance (SBA) and complications such as pre-eclampsia, hemorrhage, puerperal sepsis, among other indirect obstetrics causes have been cited as contributor to the high MMR in SSA [11–15].

Literatures excel at identifying the facilitators of maternal healthcare utilization in SSA region [16–19]. This ranges from rural to urban in the western, eastern, central, and southern Africa and includes ANC, SBA, and postnatal care (PNC), as well as the maternity gamut of care, institutional, and non-institutional delivery. Identified predictors are socio-demographics, obstetrics, and other health-related characteristics such as place of residence, maternal age, educational level, media access, healthcare decision, unwanted pregnancy, distance to health facility, socio-economic status, parity, ANC, and SBA use [20–26].

Though studies opined on institutional and non-institutional delivery but, very few have examined the combined distribution and predictors in the large SSA population [25, 26]. We are not aware of any study that focuses on evaluating non-institutional delivery outcome across multiple countries in the SSA region. Yahya et al., only compared home births in Ethiopia and Nigeria [27]. Also, there is limited evidence on observational studies on the connection between non-institutional delivery and maternal mortality in Africa [28]. Thus, our study does not only report the foremost evaluation of non-institutional delivery in lower and middle income SSA countries, but also provide fact to support strategy to bridge the regional differences in institutional delivery and improve the connected maternal mortality spike in Africa while expanding on the body of knowledge regarding non-institutional delivery domination in Sub-Saharan African countries.

Hence, we exclusively investigated the pooled country prevalence and determinants of non-institutional delivery in SSA countries, assessed the delivery gap in the rural and urban SSA communities and examined the impact of non-institutional delivery on maternal mortality and the lifetime risk of maternal death in SSA, to answer the following research questions: what is the gap in prevalence of institutional versus non-institutional delivery in urban and rural SSA; what are the socio-demographics, obstetrics and country-level factors associated with non-institutional delivery; any correlation between non-institutional delivery and maternal mortality/lifetime risk of maternal death in low and middle income SSA countries. We anticipated that findings from this study will provide a pooled estimate of non-institutional delivery in Africa, inform on the disparity in the urban–rural communities and provide an evidence-based programming strategy to guide scale-up of intervention targeted towards reducing maternal mortality spike in African region.

## Methods

### Study design and settings

**Fig. 1:**
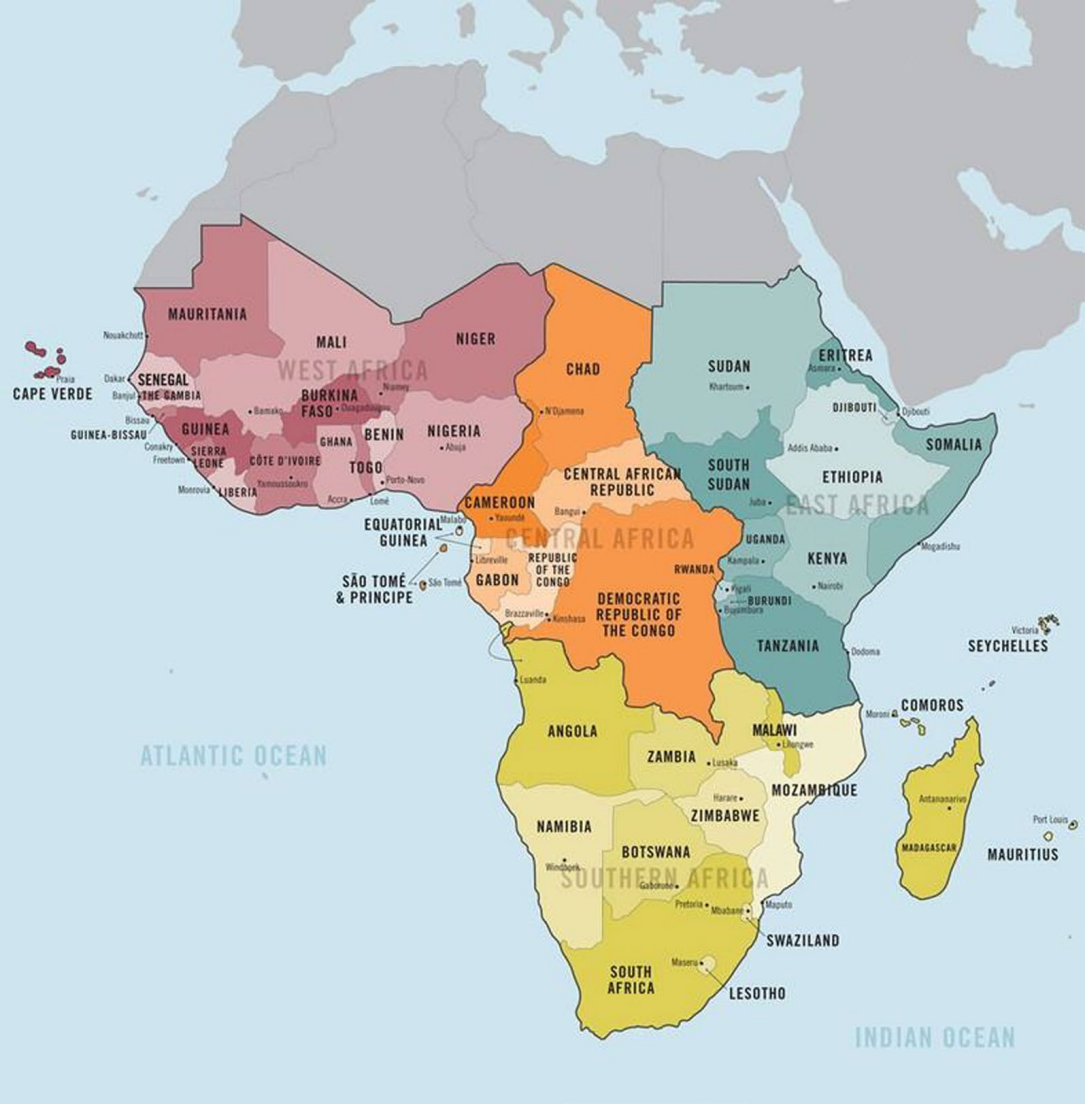
Map of Africa showing the Countries in the Sub-Saharan African Region

This study is an analysis of multi-country cross-sectional population-based surveys conducted in SSA between 2014 and 2021 by the DHS. The DHS is often conducted in five years interval and cut across the lower-middle-income countries including the sub-Saharan African countries. The SSA comprises of up to 46 countries with over a billion population and the one-fourth of the population is dominated by the reproductive age women 15–49 years [29]. Geographically, the SSA countries are in western, central, eastern, and southern Africa as shown in the study area map in Fig. 1.

### Data source

The recently (2014–2021) available country survey data on women of reproductive age 15–49 years (individual recode) was pulled from the open repository of the demographic health survey across 25 sub-Saharan Africa countries. The survey information can be accessed via the link https://dhsprogram.com

### Data collection, sampling and participants

The study population comprises of the women of reproductive age who had given birth to at least a child in the last five years preceding the country demographic health survey. The survey usually utilized a multistage sampling to select respondent per household, based on the individual country sampling frame as defined in the country population census. The first stage sampling is the selection of the district or local government areas, and the subsequent selection of the enumeration area (whether rural or urban) is the second stage sampling while the last stage sampling is the selection of the household within the enumeration areas cluster referred to as the primary sampling unit (PSU). The demographic health survey had documented the sampling strategy in the survey reports. A total of 220,865 (66,276 in urban and 154,590 in rural) women of reproductive age 15–49 years across 25 SSA countries made up the weighted sample size.

### Outcome indicators

#### Primary Outcome

The outcome of interest in this study is the type of place of delivery which is whether institutional i.e. at a health facility or non-institutional i.e. at a place other than a healthcare facility (this includes homes, faith-based, other homes i.e. friend/parent homes). Outcome variable was extracted from the response to the question “Where did you have the delivery of child?” and was classified as illustrated below.$$Type\,of\,place\,of\,delivery =\left\{\begin{array}{c}1, Non{-}institutional\,delivery\,i.e.\,home/faith\\ 0, Institutional\,delivery\,i.e.\,healthcare\,facility\end{array}\right.$$

$$Type\,of\,place\,of\,delivery =\left\{\begin{array}{c}1, Non{-}institutional\,delivery\,i.e.\,home/faith\\ 0, Institutional\,delivery\,i.e.\,healthcare\,facility\end{array}\right.$$

#### Secondary outcome

Maternal mortality which measure the annual number of female deaths from any cause related to or aggravated by pregnancy or its management (excluding accidental or incidental causes) during pregnancy and childbirth or within 42 days of termination of pregnancy, irrespective of the duration and site of the pregnancy, and the lifetime risk of maternal death which assess the probability that a 15 year old will die from a maternal cause are the secondary outcome measures [3, 30]. Both can be computed as:

$$Maternal\,Mortality\,Ratio = \frac{{Number\;of\;Maternal\left( {15 - 49\;years} \right)\,death\,at\,a\,setting/period}}{{Total\;Live\;births\;at\;the\;same\;setting/period}} \times 100,000.$$

$$and$$

$$Lifetime\;Risk\;of\;Maternal\;Death = \sum\nolimits_{x} {MMRatio_{x} } \times f_{x} \times \frac{{L_{x} }}{{L_{{15}} }}$$

where; ${MMRatio}_{x}$ is the maternal mortality ratio at age x.

${f}_{x}$ is the fertility rate at age x.

${L}_{x}$ is the number of woman-years of exposure to the risk of dying from maternal cause at age x.

${L}_{15}$ is the probability that a girl will survive to age 15.

### Explanatory factors

Explanatory variables that predetermined women type of place of delivery was identified from previous literatures assessing the related outcome in SSA [7, 8, 25–27, 31]. This is based on the factors measured in the demographic health survey. These factors were classified in the domains of socio-demographics, obstetrics, and maternal health as well as community and country-level factors as described below.

#### Socio-demographic characteristics

Age-group (15–24; 25–34; 35–49 years), highest educational level (no education; primary; secondary; higher), partner’s highest education (no education; primary; secondary; higher), occupation (not currently employed; currently employed), marital status (ever married; never married), wealth status (poor; average; rich), media exposure (not exposed to media; exposed to media), sex of household head (male; female).

#### Obstetrics and health-related factors

These are factors assessing whether women were covered by health insurance (no; yes), birth order (one, two, three, four, five and above), wanted last child (wanted then; wanted later; wanted no more), healthcare decision maker (respondent alone; respondent and husband; husband/partner alone; someone else/others), sex of child at birth (male; female), antenatal care attendance (none; 1–3; 4–7; 8 and above visits), skilled birth attendant at delivery (no; yes).

#### Community and country-level factors

This includes place of residence (urban; rural) and SSA region (central; eastern; southern; western; African Island Nation). The country income (low income; lower-middle income; upper-middle income) was classified based on the recent world bank GDP threshold and poverty level per region [32].

##### Data management and statistical analysis

The twenty-five countries survey data pulled from the DHS database was merged to achieve a pooled data about reproductive aged women (15–49 years). The merged/appended data was validated to ensure variables align throughout the dataset and data completeness and consistency was also assessed through complete omission of data missing both at random and not at random to achieve a clean data for analysis.

In preparation for analysis, the cleaned and complete data following case wise deletion of missing and incomplete maternal information/data was weighted using the women weighting indices in the DHS to adjust for population heterogeneity and verified from the frequency distribution of the outcome data. Statistical analysis commenced with the descriptive analysis of the independent factors viz-a-viz the type of place of residence. Hence the frequency and percentages were the reported statistics. The dichotomized outcome (institutional -0, non-institutional-1) was graphically presented to reveal country prevalence.

Bivariate analysis was performed to show the distribution (in frequency and percentages) of the independent factors by the outcome (institutional versus non-institutional delivery) and reveal the inherent association using the Pearson chi-square test of statistical significance set at 20% (p < 0.20) and to give allowance for equal chance of variable inclusion without stringent rule in the multivariable analysis. This was performed for both the rural and urban strata. Consequently, all the variables were reported based on the Pearson chi-square test statistic as none of the subgroup had an expected frequency less than 5 and/or 20% of the expected cell count.

Pearson Correlation and simple linear regression was performed to determine the relationship between the normally distributed country NID and MMR as well as NID and LTR estimates. Hence the correlation (rho) and regression (beta) coefficients presented the magnitude of the association. The regression intercept and the R-squared was also reported to assess the explained variation in the model. The pattern of association between NID and MMR as well as NID and LTR was assessed via the linear trend showcasing the direction of relationship. The pattern of relationship was further disaggregated by low- and middle-income countries.

Subsequently, all the 17 variables significant at the bivariate level were included in the multivariable binary logistic regression model fitted to assess the factors effect (likelihood and significance) on women type of place of delivery. Hence the adjusted odds ratio (aOR) was reported. Also, the crude odds ratio (cOR) was reported to assess the independent effect on the outcome in the absence of other factors. The inferential analysis was performed by incorporating the svyset algorithm to adjust for complex survey sampling due to data weighting, clustering, and stratification. All Analysis were performed on Stata version 18 (Texas, College station, USA) at 5% level of significance (95% Confidence Interval). Inferential statistics and/or hypothesis were performed at a two-tailed test, and multiple regression model collinearity was assessed and controlled through variance inflation factor (VIF) such that factor with VIF ≤ 5 was maintained.

##### Multiple logistic regression

A multiple binary logistic regression was fitted to determine the predictors of non-institutional delivery. This is based on the binary response classification that follows a Bernoulli distribution [P($${Y}_{i}=0$$), P($${Y}_{i}=1)]$$, such that home/non-institutional delivery = 0 and facility/institutional delivery = 1 for all ith respondents. The equation producing the regression coefficients (or the exponent as odds ratio) is thus estimable under a parabolical curve rather than the straight line in the linear regression. The multivariable binary regression model which is a linear combination of the dependent term Y and independent term X is specified below.

1 $$Y_{i} \sim {\text{ Ber }}\left( {0,{ 1}} \right)$$

2 $$Y_{i} = \ln \left( {\frac{\pi }{1 - \pi }} \right) = \alpha_{0} + \alpha_{1} X_{1i} + \cdots + \alpha_{k} X_{ki} + \varepsilon$$

3 $$E\left( {Y_{i} } \right) = Z_{i} = \frac{{\exp \left( {\alpha_{0} + \alpha_{1} x_{1i} + \cdots + \alpha_{k} x_{ki} } \right)}}{{1 + \exp \left( {\alpha_{0} + \alpha_{1} x_{1i} + \cdots + \alpha_{k} x_{ki} } \right)}}$$

where: $\text{ln}\left(\frac{\pi }{1-\pi }\right)$ is the log odds (‘π’ is the probability of giving birth at home/non-institutional settings and ‘1-π’ is the probability of giving birth in a health facility/institutional settings).

${\alpha }_{0}$ is the logistic regression constant or intercept.

${\alpha }_{1}+\cdots + {\alpha }_{k}$ are the kx1 vector of regression coefficient or slopes.

${X}_{i1}+\cdots +{X}_{ik}$ are the nxk matrix of explanatory variables predicting the log odds in the model.

## Results

### Distribution of women (15–49 years) participants by urban–rural settings

A total of 220,865 participants were recruited into this study across urban (66,276 participants, 30%) and rural (154,590 participants, 70%) divides of 25 SSA countries. All participants were women of reproductive age (15–49 years), and about half (16.1% in urban vs 33.2% in rural) were middle aged women between 25 and 34 years. Majority of the women (40.5% had no formal education, and 33.5% had primary education) and their partners (35.9% had no formal education and 30.3% had primary education) had low educational attainment. Furthermore, most of the participants were currently employed (68.3%), ever married (83.6%), not exposed to media (71.8%) and not covered by health insurance (91.5%) (Table 1).

**Table 1:** Women Characteristics by Urban and Rural Distribution in Sub-Saharan Africa. Source: Demographic Health Survey (2014–2021)

Maternal Characteristics	Urban Weighted n (%) (N = 66,276)	Rural Weighted n (%) (N = 154,590)	Overall Weighted n (%) (N = 220,865)
Age			
15–24 years	15,017 (6.8)	42,716 (19.3)	57,733 (26.1)
25–34 years	35,555 (16.1)	73,369 (33.2)	108,925 (49.3)
35–49 years	15,704 (7.1)	38,504 (17.4)	54,208 (24.5)
Highest Educational Level			
No education	16,140 (7.3)	73,320 (33.2)	89,460 (40.5)
Primary	17,379 (7.9)	56,552 (25.6)	73,930 (33.5)
Secondary	26,392 (12)	22,876 (10.4)	49,268 (22.3)
Higher	6365 (2.9)	1842 (0.8)	8207 (3.7)
Partner’s Highest Education			
No education	13,748 (6.2)	65,509 (29.7)	79,257 (35.9)
Primary	13,710 (6.2)	53,166 (24.1)	66,876 (30.3)
Secondary	28,091 (12.7)	30,982 (14)	59,073 (26.8)
Higher	10,727 (4.9)	4932 (2.2)	15,659 (7.1)
Occupation			
Not Currently employed	24,102 (10.9)	46,032 (20.8)	70,134 (31.8)
Currently employed	42,173 (19.1)	108,558 (49.2)	150,731 (68.3)
Marital Status			
Ever Married	53,529 (24.2)	131,018 (59.3)	184,547 (83.6)
Never Married	12,747 (5.8)	23,572 (10.7)	36,319 (16.4)
Wealth Status			
Poor	7555 (3.4)	91,572 (41.5)	99,127 (44.9)
Average	9878 (4.5)	34,657 (15.7)	44,535 (20.2)
Rich	48,842 (22.1)	28,361 (12.8)	77,203 (35)
Media exposure			
Not exposed to media	30,721 (13.9)	127,896 (57.9)	158,617 (71.8)
Exposed to media	35,555 (16.1)	26,694 (12.1)	62,248 (28.2)
Sex of household head			
Male	56,513 (25.6)	135,185 (61.2)	191,698 (86.8)
Female	9763 (4.4)	19,405 (8.8)	29,168 (13.2)
Covered by health insurance			
No	59,123 (26.8)	143,025 (64.8)	202,148 (91.5)
Yes	7153 (3.2)	11,565 (5.2)	18,718 (8.5)
Birth Order			
One	15,044 (6.8)	27,982 (12.7)	43,027 (19.5)
Two	15,280 (6.9)	27,821 (12.6)	43,101 (19.5)
Three	12,168 (5.5)	24,525 (11.1)	36,693 (16.6)
Four	8564 (3.9)	20,909 (9.5)	29,473 (13.3)
Five and above	15,219 (6.9)	53,352 (24.2)	68,571 (31.1)
Wanted Last Child			
Wanted then	51,757 (23.4)	121,970 (55.2)	173,726 (78.7)
Wanted later	11,264 (5.1)	24,367 (11)	35,632 (16.1)
Wanted no more	3255 (1.5)	8253 (3.7)	11,508 (5.2)
Healthcare Decision maker			
Respondent alone	12,304 (5.6)	23,656 (10.7)	35,960 (16.3)
Respondent and Husband	27,902 (12.6)	58,244 (26.4)	86,146 (39)
Husband/partner alone	25,824 (11.7)	71,782 (32.5)	97,606 (44.2)
Someone else/Others	246 (0.1)	907 (0.4)	1153 (0.5)
Sex of child			
Male	33,737 (15.3)	78,530 (35.6)	112,267 (50.8)
Female	32,538 (14.7)	76,060 (34.4)	108,598 (49.2)
Antenatal Care Attendance			
None	22,450 (10.2)	65,032 (29.4)	87,482 (39.6)
1–3 visits	20,746 (9.4)	62,127 (28.1)	82,873 (37.5)
4–7 visits	16,419 (7.4)	22,979 (10.4)	39,398 (17.8)
8 and above	6661 (3.0)	4451 (2.0)	11,112 (5.0)
Skilled birth attendant on delivery			
No	23,218 (10.5)	94,005 (42.6)	117,224 (53.1)
Yes	43,057 (19.5)	60,584 (27.4)	103,642 (46.9)
Country Income			
Low income	26,335 (11.9)	92,953 (42.1)	119,287 (54.0)
Lower Middle income	39,396 (17.8)	61,432 (27.8)	100,829 (45.7)
Upper Middle income	545 (0.3)	204 (0.1)	749 (0.3)
Region			
Central Africa	2220 (1)	16,346 (7.4)	18,566 (8.4)
East Africa	7299 (3.3)	20,831 (9.4)	28,129 (12.7)
Southern Africa	12,824 (5.8)	26,396 (12)	39,220 (17.8)
West Africa	42,447 (19.2)	82,931 (37.6)	125,378 (56.8)
African Island Nation	1486 (0.7)	8086 (3.7)	9572 (4.3)

n-Counts; %-Percent

About 20% of the women equally had one and two previous births respectively. More than four-fifth (78.7%) wanted the current pregnancy while the combined 21.3% either wanted the pregnancy later or no more. Majority (44.2%) of healthcare decision was made by the husband/partner alone. The proportion of male (50.8%) is almost the proportion of female child at birth (49.2%). About 18% (39,398) of women achieved 4–7 ANC visits while only 5.0% (11,112) attended ANC in 8 or more visits. More than half (54.0%) of the women resides in a low income SSA countries while very few (< 1%) lives in an upper middle-income country. About 57% (125,378) are Western Africa countries, 17.8% are Southern Africa, 12.7% Eastern Africa and 8.4% Central Africa and as few as 4.3% of the women are from African Island nation (Table 1).

### Country prevalence of institutional versus non-institutional delivery

**Fig. 2:**
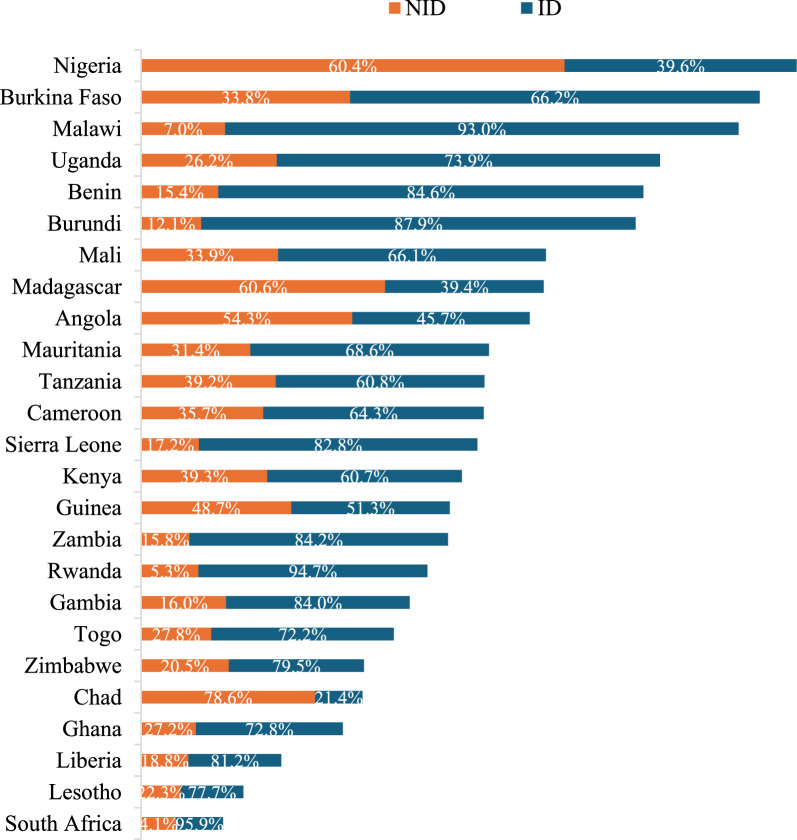
Distribution of prevalence among Sub-Saharan African Countries by Institutional and Non-institutional Delivery

Figure 2 shows the prevalence of institutional versus non-institutional delivery in SSA. NID was highest in Chad with about 78.6% prevalence, then Madagascar (60.6%) and Nigeria (60.4%). NID prevalence is lowest in South Africa (4.1%). Prevalence of institutional birth is equivalently highest in South Africa (95.9%), followed by Rwanda (94.7%) and Malawi (93.0%) but lowest in Chad (21.4%).

### Bivariate association between NID and women characteristics

Overall, the prevalence of NID was highest among women in rural areas (40.4%), women without formal education (48.6%), in poor wealth quintiles (48.7%), without ANC (47.2%) and a skilled birth attendant present on delivery (60.3%) and in women from African Island Nation (60.6%). In the urban area, NID was highest among women without skilled birth attendant at delivery (43.9%) followed by women in poor wealth quintiles (40.3%) and those from African Island Nation (38.9%). Whereas in the rural, NID was as high as 64.6% among women in African Island, 64.4% in women without SBA at birth and 54.5% among women without ANC visit. All the factors studied are associated with the NID in rural and urban areas and in both rural and urban combined at p < 0.001 under the bivariate chi-square analysis (Table 2).

**Table 2:** Bivariate Chi-square Association between Women Delivery Choice and their Characteristics in Urban and Rural Sub-Saharan Africa. Source: Demographic Health Survey (2014–2021)

	Urban		Rural		Overall	
Maternal Characteristics	Institutional N = 54,612 [82.4%) n (row %)	Non-Institutional N = 11,664 [17.6%] n (row %)	Institutional N = 92,079 [59.6%] n (row %)	Non-Institutional N = 62,511 [40.4%] n (row %)	Institutional N = 146,691 [66.4%] n (row %)	Non-Institutional N = 74,175 [33.6%] n (row %)
Individual Variables						
Age	68.198 (< 0.001^b^)	124.411(< 0.001^b^)	131.540 (< 0.001^b^)			
15–24 years	12,126 (80.8)	2891 (19.2)	26,116 (61.1)	16,600 (38.9)	38,242 (66.2)	19,491 (33.8)
25–34 years	29,697 (83.5)	5859 (16.5)	43,800 (59.7)	29,569 (40.3)	73,497 (67.5)	35,428 (32.5)
35–49 years	12,789 (81.4)	2914 (18.6)	22,163 (57.6)	16,341 (42.4)	34,952 (64.5)	19,256 (35.5)
Highest Educational Level	3.5e + 03 (< 0.001^b^)	1.0e + 04 (< 0.001^b^)	1.9e + 04 (< 0.001^b^)			
No education	11,280 (69.9)	4860 (30.1)	34,677 (47.3)	38,643 (52.7)	45,956 (51.4)	43,503 (48.6)
Primary	13,914 (80.1)	3464 (19.9)	38,113 (67.4)	18,439 (32.6)	52,027 (70.4)	21,903 (29.6)
Secondary	23,344 (88.5)	3048 (11.5)	17,597 (76.9)	5279 (23.1)	40,940 (83.1)	8328 (16.9)
Higher	6074 (95.4)	291 (4.6)	1692 (91.9)	150 (8.1)	7766 (94.6)	441 (5.4)
Partner’s Highest Education	2.1e + 03 (< 0.001^b^)	7.3e + 03 (< 0.001^b^)	1.4e + 04 (< 0.001^b^)			
No education	9885 (71.9)	3863 (28.1)	31,318 (47.8)	34,192 (52.2)	41,203 (52.0)	38,055 (48.0)
Primary	10,998 (80.2)	2712 (19.8)	34,850 (65.6)	18,316 (34.5)	45,848 (68.6)	21,028 (31.4)
Secondary	24,000 (85.4)	4091 (14.6)	22,104 (71.3)	8878 (28.7)	46,104 (78.1)	12,969 (21.9)
Higher	9729 (90.7)	998 (9.3)	3807 (77.2)	1125 (22.8)	13,536 (86.4)	2123 (13.6)
Occupation	17.857 (< 0.001^b^)	570.951(< 0.001^b^)	265.398 (< 0.001^b^)			
Not Currently employed	19,883 (82.5)	4220 (17.5)	25,369 (55.1)	20,663 (44.9)	45,252 (64.5)	24,883 (35.5)
Currently employed	34,729 (82.4)	7444 (17.6)	66,710 (61.5)	41,848 (38.6)	101,439 (67.3)	49,292 (32.7)
Marital Status	73.558 (< 0.001^b^)	87.489 (< 0.001^b^)	64.229 (< 0.001^b^)			
Ever Married	44,460 (83.1)	9069 (16.9)	77,148 (58.9)	53,871 (41.1)	121,607 (65.9)	62,939 (34.1)
Never Married	10,152 (79.6)	2595 (20.4)	14,931 (63.3)	8640 (36.7)	25,083 (69.1)	11,235 (30.9)
Wealth Status	5.5e + 03 (< 0.001^b^)	8.3e + 03 (< 0.001^b^)	2.2e + 04 (< 0.001^b^)			
Poor	4508 (59.7)	3047 (40.3)	46,326 (50.6)	45,246 (49.4)	50,834 (51.3)	48,293 (48.7)
Average	6841 (69.3)	3037 (30.8)	23,077 (66.6)	11,580 (33.4)	29,918 (67.2)	14,617 (32.8)
Rich	43,262 (88.6)	5580 (11.4)	22,676 (80.0)	5685 (20.0)	65,938 (85.4)	11,265 (14.6)
Media exposure	1.9e + 03 (< 0.001^b^)	2.6e + 03 (< 0.001^b^)	8.9e + 03 (< 0.001^b^)			
Not exposed to media	23,214 (75.6)	7507 (24.4)	72,373 (56.6)	55,523 (43.4)	95,587 (60.3)	63,030 (39.7)
Exposed to media	31,398 (88.3)	4157 (11.7)	19,706 (73.8)	6988 (26.2)	51,104 (82.1)	11,145 (17.9)
Sex of household head	57.934 (< 0.001^b^)	354.080 (< 0.001^b^)	553.409 (< 0.001^b^)			
Male	46,218 (81.8)	10,295 (18.2)	79,178 (58.6)	56,007 (41.4)	125,396 (65.4)	66,302 (34.6)
Female	8394 (86.0)	1369 (14.0)	12,901 (66.5)	6504 (33.5)	21,295 (73.0)	7873 (27.0)
Covered by health insurance	823.902 (< 0.001^b^)	2.9e + 03 (< 0.001^b^)	4.3e + 03 (< 0.001^b^)			
No	47,850 (80.9)	11,273 (19.1)	82,240 (57.5)	60,785 (42.5)	130,090 (64.4)	72,058 (35.7)
Yes	6762 (94.5)	391 (5.5)	9839 (85.1)	1726 (14.9)	16,601 (88.7)	2117 (11.3)
Birth Order	1.4e + 03 (< 0.001^b^)	2.7e + 03 (< 0.001^b^)	5.3e + 03 (< 0.001^b^)			
One	13,357 (88.8)	1688 (11.2)	19,691 (70.4)	8291 (29.6)	33,048 (76.8)	9979 (23.2)
Two	13,146 (86.0)	2133 (14.0)	17,558 (63.1)	10,263 (36.9)	30,704 (71.2)	12,397 (28.8)
Three	10,144 (83.4)	2024 (16.6)	15,022 (61.3)	9503 (38.8)	25,166 (68.6)	11,527 (31.4)
Four	6978 (81.5)	1586 (18.5)	12,197 (58.3)	8712 (41.7)	19,175 (65.1)	10,298 (34.9)
Five and above	10,987 (72.2)	4232 (27.8)	27,611 (51.8)	25,741 (48.3)	38,598 (56.3)	29,974 (43.7)
Wanted Last Child	35.631(< 0.001^b^)	1.5e + 03 (< 0.001^b^)	1.3e + 03 (< 0.001^b^)			
Wanted then	42,484 (82.1)	9272 (17.9)	69,654 (57.1)	52,316 (42.9)	112,138 (64.6)	61,588 (35.5)
Wanted later	9477 (84.1)	1787 (15.9)	16,839 (69.1)	7529 (30.9)	26,316 (73.9)	9315 (26.1)
Wanted no more	2650 (81.4)	605 (18.6)	5586 (67.7)	2666 (32.3)	8237 (71.6)	3271 (28.4)
Healthcare Decision maker	706.237 (< 0.001^b^)	2.7e + 03 (< 0.001^b^)	3.9e + 03 (< 0.001^b^)			
Respondent alone	10,413 (84.6)	1892 (15.4)	15,413 (65.2)	8243 (34.9)	25,825 (71.8)	10,135 (28.2)
Respondent and Husband	24,016 (86.1)	3885 (13.9)	38,664 (66.4)	19,581 (33.6)	62,680 (72.8)	23,466 (27.2)
Husband/partner alone	19,981 (77.4)	5843 (22.6)	37,444 (52.2)	34,338 (47.8)	57,425 (58.8)	40,181 (41.2)
Someone else/Others	201 (81.9)	45 (18.1)	559 (61.6)	348 (38.4)	760 (65.9)	393 (34.1)
Sex of child	17.567 (< 0.001^b^)	24.309 (< 0.001^b^)	40.665 (< 0.001^b^)			
Male	28,058 (83.2)	5679 (16.8)	47,161 (60.1)	31,368 (39.9)	75,220 (67.0)	37,047 (33.0)
Female	26,553 (81.6)	5985 (18.4)	44,918 (59.1)	31,143 (40.9)	71,471 (65.8)	37,127 (34.2)
Antenatal Care Attendance	2.1e + 03 (< 0.001^b^)	9.8e + 03 (< 0.001^b^)	1.4e + 04 (< 0.001^b^)			
None	16,618 (74.0)	5833 (26.0)	29,620 (45.6)	35,412 (54.5)	46,237 (52.9)	41,245 (47.2)
1–3 visits	17,494 (84.3)	3251 (15.7)	42,007 (67.6)	20,120 (32.4)	59,501 (71.8)	23,372 (28.2)
4–7 visits	14,463 (88.1)	1956 (11.9)	16,886 (73.5)	6094 (26.5)	31,349 (79.6)	8049 (20.4)
8 and above	6037 (90.6)	624 (9.4)	3566 (80.1)	885 (19.9)	9604 (86.4)	1509 (14.6)
Skilled birth attendant on delivery	1.6e + 04 (< 0.001^b^)	6.2e + 04 (< 0.001^b^)	8.3e + 04 (< 0.001^b^)			
No	13,030 (56.1)	10,189 (43.9)	33,506 (35.6)	60,499 (64.4)	46,536 (39.7)	70,688 (60.3)
Yes	41,582 (96.6)	1475 (3.4)	58,573 (96.7)	2011 (3.3)	100,155 (96.6)	3487 (3.4)
Country-level variables						
Country Income	1.0e + 03 (< 0.001^b^)	5.9e + 03 (< 0.001^b^)	4.3e + 03 (< 0.001^b^)			
Low income	23,316 (88.5)	3019 (11.5)	63,009 (67.8)	29,944 (32.2)	86,324 (72.4)	32,963 (27.6)
Lower Middle income	30,767 (78.1)	8629 (21.9)	28,881 (47.0)	32,552 (53.0)	59,648 (59.2)	41,181 (40.8)
Upper Middle income	529 (97.1)	16 (2.9)	189 (92.7)	15 (7.3)	718 (95.9)	31 (4.1)
Region	1.1e + 03 (< 0.001^b^)	1.2e + 04 (< 0.001^b^)	1.1e + 04 (< 0.001^b^)			
Central Africa	2168 (97.6)	53 (2.4)	14,612 (89.4)	1734 (10.6)	16,780 (90.4)	1787 (9.6)
East Africa	6261 (85.8)	1037 (14.2)	12,442 (59.7)	8389 (40.3)	18,703 (66.5)	9426 (33.5)
Southern Africa	10,564 (82.4)	2259 (17.6)	19,829 (75.1)	6567 (24.9)	30,394 (77.5)	8826 (22.5)
West Africa	34,711 (81.8)	7737 (18.2)	42,334 (51.0)	40,597 (49.0)	77,044 (61.5)	48,334 (38.6)
African Island Nation	908 (61.1)	578 (38.9)	2862 (35.4)	5224 (64.6)	3770 (39.4)	5802 (60.6)

^b^–significant at 20%; n-Counts; %-Percent

### Predictors of non-institutional delivery in urban–rural SSA

The results in Table 3 show that the odds ratio of NID across all 25 SSA countries was lower among women aged 25–34 years (aOR: 0.65; 95% CI: 0.63–0.67) and women aged 35–49 years (aOR: 0.57, 95% CI: 0.54–0.60). This finding was consistent across rural and urban areas. Overall, the odds of NID significantly reduced with a lower level of formal education (aOR: 0.92; 95% CI: 0.89–0.95 for primary education; aOR: 0.81; 95% CI: 0.77–0.84 for secondary education, and aOR: 0.75; 95% CI: 0.66–0.86 for higher education). Similar findings were observed in rural areas.

**Table 3:** Predictors “Odds (95% Confidence Interval)” of Non-institutional Delivery by Urban and Rural Locations in Sub-Saharan Africa. Source: Demographic Health Survey (2014–2021)

Variable	Non-Institutional Delivery					
	Urban		Rural		Overall	
	cOR (95% CI)	aOR (95% CI)	cOR (95% CI)	aOR (95% CI)	cOR (95% CI)	aOR (95% CI)
Individual variables						
Age						
15–24 years	Reference					
25–34 years	0.82 (0.78, 0.86)	0.59 (0.54, 0.63) *	1.08 (1.05, 1.1)	0.69 (0.66, 0.72) *	0.97 (0.95, 0.99)	0.65 (0.63, 0.67) *
35–49 years	0.92 (0.87, 0.97)	0.44 (0.40, 0.48) *	1.17 (1.14, 1.2)	0.64 (0.6, 0.67) *	1.09 (1.07, 1.12)	0.57 (0.54, 0.6) *
Highest educational level						
No education	Reference					
Primary	0.55 (0.53, 0.58)	0.97 (0.90, 1.04)	0.43 (0.42, 0.44)	0.91 (0.88, 0.95) *	0.44 (0.43, 0.45)	0.92 (0.89, 0.95) *
Secondary	0.28 (0.27, 0.3)	0.84 (0.77, 0.91) *	0.27 (0.26, 0.28)	0.8 (0.76, 0.85) *	0.22 (0.21, 0.22)	0.81 (0.77, 0.84) *
Higher	0.1 (0.08, 0.11)	0.73 (0.61, 0.87) *	0.08 (0.07, 0.1)	0.89 (0.71, 1.11)	0.06 (0.05, 0.06)	0.75 (0.66, 0.86) *
Partner’s highest education						
No education	Reference					
Primary	0.58 (0.55, 0.61)	0.88 (0.81, 0.95) *	0.48 (0.47, 0.49)	0.89 (0.86, 0.93) *	0.49 (0.48, 0.5)	0.89 (0.86, 0.92) *
Secondary	0.41 (0.39, 0.43)	1.13 (1.05, 1.23) *	0.37 (0.36, 0.38)	0.99 (0.95, 1.04)	0.31 (0.3, 0.32)	1.02 (0.98, 1.06)
Higher	0.23 (0.22, 0.25)	1.41 (1.26, 1.58) *	0.27 (0.25, 0.29)	1.61 (1.44, 1.79) *	0.17 (0.16, 0.18)	1.43 (1.32, 1.54) *
Occupation						
Not currently employed	Reference					
Currently employed	0.92 (0.88, 0.95)	0.69 (0.66, 0.73) *	0.77 (0.75, 0.79)	0.65 (0.62, 0.67) *	0.86 (0.84, 0.87)	0.66 (0.65, 0.68) *
Marital status						
Ever married	Reference					
Never married	1.24 (1.18, 1.3)	0.82 (0.76, 0.88) *	0.87 (0.85, 0.9)	1.05 (1, 1.09) *	0.91 (0.88, 0.93)	0.95 (0.92, 0.98) *
Wealth Status						
Poor	Reference					
Average	0.58 (0.55, 0.61)	0.85 (0.79, 0.92) *	0.51 (0.5, 0.52)	0.56 (0.54, 0.59) *	0.5 (0.49, 0.51)	0.6 (0.58, 0.62) *
Rich	0.18 (0.17, 0.19)	0.34 (0.32, 0.37) *	0.27 (0.26, 0.28)	0.36 (0.34, 0.38) *	0.19 (0.18, 0.19)	0.3 (0.29, 0.31) *
Media exposure						
Not exposed to media	Reference					
Exposed to media	0.4 (0.39, 0.42)	0.71 (0.67, 0.75) *	0.47 (0.46, 0.49)	0.7 (0.68, 0.73) *	0.34 (0.34, 0.35)	0.68 (0.66, 0.7) *
Sex of household head						
Male	Reference					
Female	0.8 (0.75, 0.85)	0.71 (0.67, 0.75) *	0.74 (0.72, 0.77)	1 (0.95, 1.04)	0.73 (0.71, 0.75)	0.98 (0.94, 1.01)
Covered by health insurance						
No	Reference					
Yes	0.27 (0.25, 0.3)	0.79 (0.71, 0.89) *	0.27 (0.26, 0.28)	1.09 (1.01, 1.17) *	0.25 (0.24, 0.26)	1 (0.93, 1.06)
Birth order						
One	Reference					
Two	1.35 (1.26, 1.44)	1.73 (1.58, 1.88) *	1.38 (1.34, 1.43)	1.68 (1.6, 1.77) *	1.35 (1.31, 1.4)	1.69 (1.62, 1.77) *
Three	1.58 (1.47, 1.69)	2.28 (2.08, 2.51) *	1.51 (1.45, 1.56)	2.1 (1.98, 2.22) *	1.52 (1.48, 1.57)	2.15 (2.05, 2.25) *
Four	1.81 (1.68, 1.94)	2.87 (2.58, 3.19) *	1.69 (1.63, 1.76)	2.4 (2.26, 2.55) *	1.77 (1.72, 1.83)	2.55 (2.42, 2.69) *
Five and above	2.88 (2.71, 3.07)	4.47 (4.03, 4.97) *	2.16 (2.09, 2.22)	3.04 (2.86, 3.22) *	2.48 (2.42, 2.55)	3.39 (3.22, 3.57) *
Wanted last child						
Wanted then	Reference					
Wanted later	0.84 (0.8, 0.89)	0.89 (0.83, 0.96) *	0.6 (0.58, 0.61)	0.9 (0.87, 0.94) *	0.65 (0.63, 0.66)	0.89 (0.86, 0.93) *
Wanted no more	1.01 (0.93, 1.11)	0.91 (0.80, 1.02)	0.64 (0.61, 0.67)	1.08 (1, 1.16) *	0.72 (0.69, 0.75)	1.01 (0.95, 1.08)
Healthcare decision maker						
Respondent alone	Reference					
and Husband	0.86 (0.81, 0.91)	0.81 (0.75, 0.88) *	0.97 (0.94, 1)	0.92 (0.88, 0.96) *	0.97 (0.94, 0.99)	0.9 (0.86, 0.93) *
Husband/partner alone	1.54 (1.45, 1.63)	0.94 (0.87, 1.01)	1.67 (1.62, 1.72)	1 (0.95, 1.05)	1.72 (1.67, 1.76)	0.99 (0.95, 1.03)
Someone else/others	1.23 (0.9, 1.67)	0.94 (0.63, 1.41)	1.14 (1, 1.31)	0.82 (0.69, 0.99) *	1.27 (1.12, 1.43)	0.85 (0.72, 1.01)
Sex of child						
Male	Reference					
Female	1.09 (1.05, 1.13)	1.09 (1.04, 1.15) *	1.05 (1.03, 1.07)	1.06 (1.03, 1.09) *	1.06 (1.04, 1.08)	1.07 (1.04, 1.09) *
Antenatal care attendance						
None	Reference					
1–3 visits	0.48 (0.46, 0.5)	0.43 (0.41, 0.46) *	0.41 (0.4, 0.42)	0.43 (0.41, 0.44) *	0.44 (0.43, 0.44)	0.43 (0.42, 0.44) *
4–7 visits	0.36 (0.34, 0.38)	0.47 (0.44, 0.51) *	0.3 (0.29, 0.31)	0.38 (0.36, 0.39) *	0.29 (0.28, 0.3)	0.40 (0.38, 0.41) *
8 and above	0.26 (0.24, 0.29)	0.62 (0.55, 0.69) *	0.2 (0.18, 0.21)	0.55 (0.49, 0.61) *	0.17 (0.16, 0.18)	0.56 (0.52, 0.61) *
Skilled birth attendant on delivery						
No	Reference					
Yes	0.05 (0.04, 0.05)	0.05 (0.05, 0.05) *	0.02 (0.02, 0.02)	0.02 (0.02, 0.02) *	0.02 (0.02, 0.02)	0.02 (0.02, 0.02) *
Country Income						
Low income	4.57 (2.62, 7.94)	1.10 (0.71, 1.70)	6.77 (4.20, 10.93)	1.07 (0.58, 1.95)	8.34 (5.81, 11.96)	1.36 (0.74, 2.53)
Lower middle income	8.93 (5.14, 15.53)	1.63 (1.05, 2.51) *	14.99 (9.29, 24.20)	1.64 (0.90, 3.00)	14.76 (10.29, 21.16)	1.93 (1.04, 3.57) *
Upper middle income	Reference					
Region						
Central Africa	Reference					
East Africa	7.27 (5.81, 9.11)	7.19 (5.62, 9.21) *	5.86 (5.53, 6.21)	9.44 (8.76, 10.17) *	5.39 (5.1, 5.7)	8.6 (8.02, 9.21) *
Southern Africa	8.21 (6.59, 10.24)	7.31 (5.73, 9.32) *	2.8 (2.64, 2.97)	8.78 (8.11, 9.5) *	2.89 (2.74, 3.05)	7.36 (6.86, 7.9) *
West Africa	8.78 (7.07, 10.91)	6.54 (5.17, 8.28) *	7.4 (7.02, 7.81)	6.63 (6.21, 7.09) *	6 (5.7, 6.31)	6.19 (5.81, 6.58) *
African Island Nation	23.46 (18.55, 29.66)	17.88 (13.86, 23.06) *	16.61 (15.49, 17.82)	16.04 (14.76, 17.44) *	15.35 (14.39, 16.37)	15.41 (14.27, 16.65) *

^*^-significant at 5%; cOR-Crude Odds Ratio; aOR-Adjusted Odds Ratio

Among all women, including those in urban and rural areas, the odds of non-institutional delivery (NID) were lower for currently employed women compared to unemployed women. The adjusted odds ratios (aOR) were 0.66 for all women (95% CI: 0.65–0.68), 0.69 for urban residents (95% CI: 0.66–0.73), and 0.65 for rural residents (95% CI: 0.62–0.67). Rural resident women who were exposed to media had lower odds of NID than women not exposed to media (aOR: 0.70, 95% CI: 0.68–0.73). Across both divides, the odds of NID were lower among women who had antenatal care attendance and women who had a skilled birth attendant on child’s delivery (Table 3).

On the other hand, women domiciled in urban areas of lower middle-income countries had higher odds of NID compared to women resident in upper middle-income countries (aOR: 1.63, 95% CI: 1.05–2.51). Compared to Central African region, the odds of NID was also higher in East Africa (aOR: 8.06; 95% CI: 8.02–9.21), Southern Africa (aOR: 7.36; 95% CI: 6.86–7.90), West Africa (aOR: 6.19; 95% CI: 5.81–6.58), and African Island Nation (aOR: 15.41; 95% CI: 14.27–16.65). this is consistent across the urban and rural divides of the SSA. The odds of NID significantly increase with increase in women birth order and this pattern of increase in odds as birth order increase was observed in the stratification of urban and rural.

### Country-level predictors of non-institutional delivery

Table 4 shows the country-level prevalence and predictors of NID by rural and urban divide. NID prevalence was prominent in rural SSA and highest in rural Burundi (97.3%) and closely followed by rural Burkina Faso (97.1%). NID rate is as high as 96.3% in rural Mali and 96.2% in rural Rwanda. On the other hand, NID rate in rural SSA is low and above average in urban South Africa (51.7%). Also, about 44%, 37.6% and 37.1% of delivery in urban Gambia, Liberia and Angola are non-institutional respectively. Compared to South Africa, odds of NID was significantly higher in other SSA countries with highest likelihood observed in Chad (OR = 88.25, 95%CI = 61.15–127.35), next by Madagascar (OR = 33.84, 95%CI = 23.54–48.64) and Nigeria (OR = 31.08, 95%CI = 21.66–44.59). The odds of NID was also highest in rural (OR = 102.91, 95%CI = 63.2–167.59) and urban Chad (OR = 33.23, 95%CI = 18.89–58.48) and in excess of about 65% in rural Angola (OR = 64.9, 95%CI = 39.98–105.47) compared to South Africa.

**Table 4:** Country-level Predictors “Odds (95% Confidence Interval)” of Non-institutional Delivery by Urban and Rural Locations in Sub-Saharan Africa. Source: Demographic Health Survey (2014–2021)

Country		Urban		Rural		Overall
	n (%)	OR (95% CI)	n (%)	OR (95% CI)	n (%)	OR (95% CI)
Angola	1861 (37.1)	17.7 (10.16, 30.84) *	3157 (62.9)	64.94 (39.98, 105.47) *	5018 (100.0)	28.24 (19.65, 40.59) *
Benin	426 (23.2)	3.25 (1.85, 5.7) *	1409 (76.8)	3.39 (2.09, 5.49) *	1836 (100.0)	3.92 (2.73, 5.65) *
Burkina Faso	144 (2.9)	2.93 (1.66, 5.17) *	4825 (97.1)	8.27 (5.12, 13.37) *	4969 (100.0)	9.68 (6.74, 13.91) *
Burundi	39 (2.7)	1.19 (0.65, 2.18)	1387 (97.3)	2.16 (1.34, 3.5) *	1426 (100.0)	2.85 (1.98, 4.11) *
Cameroon	413 (14.2)	4.48 (2.55, 7.86) *	2493 (85.8)	12.71 (7.85, 20.59) *	2906 (100.0)	10.38 (7.21, 14.93) *
Chad	473 (11.4)	33.23 (18.89, 58.48) *	3662 (88.6)	102.91 (63.2, 167.59) *	4135 (100.0)	88.25 (61.15, 127.35) *
Gambia	448 (43.9)	4.07 (2.31, 7.14) *	573 (56.1)	4.99 (3.08, 8.09) *	1021 (100.0)	5.3 (3.68, 7.64) *
Ghana	192 (14.7)	3.86 (2.18, 6.83) *	1113 (85.3)	10.92 (6.73, 17.71) *	1305 (100.0)	9.48 (6.58, 13.67) *
Guinea	317 (8.9)	6.44 (3.66, 11.32) *	3258 (91.1)	23.36 (14.43, 37.81) *	3575 (100.0)	21.57 (15, 31.01) *
Kenya	488 (16.3)	9.21 (5.26, 16.12) *	2512 (83.7)	18.75 (11.58, 30.34) *	3000 (100.0)	18.15 (12.63, 26.09) *
Lesotho	57 (10.5)	2.7 (1.44, 5.05) *	485 (89.5)	5.76 (3.54, 9.4) *	542 (100.0)	6.76 (4.66, 9.8) *
Liberia	236 (37.6)	4.84 (2.72, 8.62) *	391 (62.4)	4.46 (2.74, 7.26) *	627 (100.0)	5.62 (3.88, 8.12) *
Madagascar	578 (10.0)	19.43 (11.1, 34.03) *	5224 (90.0)	28.34 (17.52, 45.84) *	5802 (100.0)	33.84 (23.54, 48.64) *
Malawi	57 (5.8)	0.94 (0.52, 1.72)	940 (94.2)	1.03 (0.64, 1.67)	997 (100.0)	1.42 (0.98, 2.04)
Mali	122 (3.8)	6.52 (3.72, 11.46) *	3136 (96.3)	10.19 (6.3, 16.49) *	3258 (100.0)	12.06 (8.39, 17.33) *
Mauritania	179 (6.9)	2.55 (1.44, 4.49) *	2417 (93.1)	10.3 (6.36, 16.67) *	2597 (100.0)	8.64 (6, 12.42) *
Nigeria	4400 (23.1)	18.27 (10.5, 31.78) *	14,665 (76.9)	34.2 (21.17, 55.25) *	19,065 (100.0)	31.08 (21.66, 44.59) *
Rwanda	14 (3.9)	0.36 (0.17, 0.75) *	347 (96.2)	0.93 (0.57, 1.52)	361 (100.0)	1.16 (0.8, 1.69)
Sierra Leone	271 (19.7)	3.54 (2, 6.25) *	1102 (80.3)	3.52 (2.17, 5.7) *	1373 (100.0)	4.49 (3.11, 6.46) *
South Africa	16 (51.7)	Reference	15 (48.3)		31 (100.0)	
Tanzania	280 (8.7)	4.74 (2.68, 8.36) *	2920 (91.3)	12.21 (7.55, 19.75) *	3200 (100.0)	13.76 (9.57, 19.78) *
Togo	116 (6.9)	1.74 (0.97, 3.15)	1553 (93.1)	9.82 (6.06, 15.9) *	1668 (100.0)	9.73 (6.76, 14.01) *
Uganda	270 (8.4)	3.67 (2.08, 6.48) *	2956 (91.6)	6.07 (3.75, 9.81) *	3226 (100.0)	7.82 (5.44, 11.23) *
Zambia	171 (14.9)	2.13 (1.2, 3.81) *	981 (85.1)	3.72 (2.29, 6.03) *	1152 (100.0)	4.29 (2.97, 6.18) *
Zimbabwe	96 (8.8)	1.62 (0.9, 2.92)	990 (91.2)	4.69 (2.88, 7.61) *	1086 (100.0)	4.5 (3.12, 6.5) *

^*^-significant at 5%; OR-Odds Ratio; n-counts; %-percent

### Correlation between NID and MMR in SSA

**Fig. 3:**
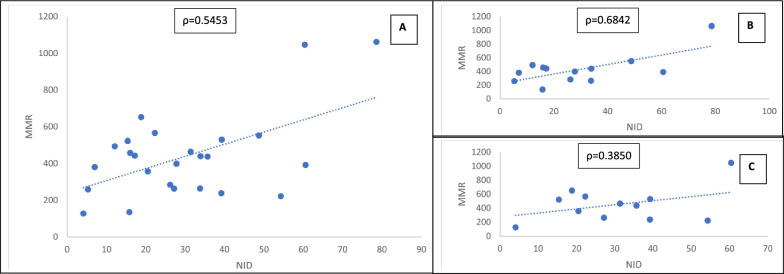
Correlation between Non-institutional Delivery (NID) and Maternal Mortality Ratio (MMR) in Sub-Saharan Africa. **A** Linear relationship between Non-institutional Delivery and Maternal Mortality Ratio in Sub-Sharan Africa. **B** Linear relationship between Non-institutional Delivery and Maternal Mortality Ratio in low-income Sub-Saharan Africa. **C** linear relationship between Non-institutional Delivery and Maternal Mortality Ratio in Middle-income Sub-Saharan Africa

Figure 3 presents the MMR pattern by NID. Overall, a sharp rise observed indicates that a positive increase in NID prevalence is associated with a steady increase in MMR. Hence a linear ascending pattern of relationship with correlation coefficient of 0.5453. This was more pronounced in low income SSA countries with a linear pattern of correlation coefficient of 0.6842 compared to the middle-income countries with correlation coefficient of 0.3850. The regression results presented in Table 5 reveal that the positive association between NID and MMR is significant (p-value = 0.0048) with 1% increase in NID leading to about 248/100000 increased in MMR and about 30% of variation in MMR was explained by the NID.

**Table 5:** Linear association between maternal mortality ratio & non-institutional delivery, lifetime risk of maternal death & non-institutional delivery and maternal mortality ratio & lifetime risk of maternal death in Sub-Saharan Africa. Source: Demographic Health Survey (2014–2021)

Coefficient	Estimate	Standard error	*t* statistic	*p*-value	95%CI
*Regression of MMR on NID*					
Intercept	241.1280	74.7787	3.2245	0.0037518	86.43–395.82
Slope	6.6043	2.1167	3.1201	0.004813	2.22–10.98
R-Squared	0.2974	196.3519			

ANOVA	DF	SS	MS	F Statistics	F-significance
Regression	1	375,317.7132	375,317.7132	9.734839	0.004813
Residual	23	886,743.7268	38,554.07508		
Total	24	1,262,061.44			

Coefficient	Estimate	Standard error	t statistic	*p*-value	95%CI
*Regression of LTR on NID*					
Intercept	0.0078	0.0042	1.8740	0.0737	-0.0008–0.0164
Slope	0.0004	0.0001	3.7268	0.0011	0.0002–0.0007
R-Squared	0.3765	0.0109			

ANOVA	DF	SS	MS	F Statistics	F-significance
Regression	1	0.0016	0.0017	13.889	0.0011
Residual	23	0.0028	0.0001		
Total	24	0.0044			

Coefficient	Estimate	Standard error	t statistic	*p*-value	95%CI
*Regression of MMR on LTR*					
Intercept	100.1198	25.8967	3.8661	0.0007	46.54–153.69
Slope	16,154.06	1041.36	15.5124	< 0.0001	13,999.8–18,308.3
R-Squared	0.9128	69.1894			

ANOVA	DF	SS	MS	F Statistics	F-significance
Regression	1	1,151,956.37	1,151,956.37	240.6338	< 0.0001
Residual	23	110,105.07	4787.18		
Total	24	1,262,061.44			

ANOVA-Analysis of Variance; DF-Degree of freedom; SS-Sum of Squares; MS-Mean Squares

NID-Non-institutional Delivery; MMR-Maternal Mortality Ratio; LTR-Lifetime Risk of Maternal Death

### Correlation between NID and LTR of maternal death in SSA

**Fig. 4:**
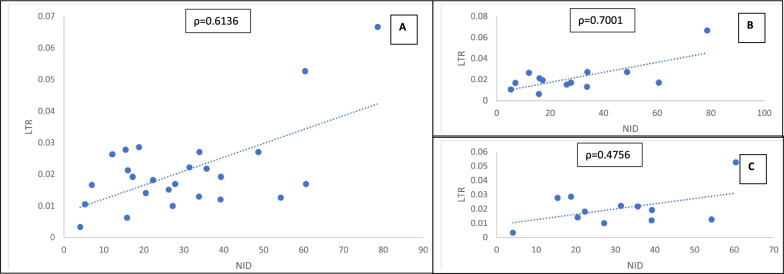
Correlation between Non-institutional Delivery (NID) and Lifetime Risk (LTR) of Maternal Death in Sub-Sharan Africa. **A** Linear relationship between Non-institutional Delivery and Lifetime Risk of Maternal Death in Sub-Sharan Africa. **B** Linear relationship between Non-institutional Delivery and Lifetime Risk of Maternal Death in Low-income Sub-Sharan Africa. **C** Linear relationship between Non-institutional Delivery and Lifetime Risk of Maternal Death in Middle-income Sub-Saharan Africa

The pattern of LTR-maternal death by NID is shown in Fig. 4. Overall, LTR-maternal death is positively associated with NID such that a positive increase in NID is associated with a corresponding increase in LTR-maternal death. Hence a linear ascending pattern of relationship with correlation coefficient of 0.6136 was observed. This was more prominent in low income (rho = 0.7001) than the middle income (rho = 0.4756) SSA countries. The corresponding linear regression presented in Table 5 further ascertain the significant (p-value = 0.0011) relationship between NID and LTR with 1% rise in NID leading to about 8.2/1000 increase in LTR-maternal death and the NID data explained about 38% of the variation in the LTR-maternal death.

### Correlation between LTR of maternal death and MMR in SSA

**Fig. 5:**
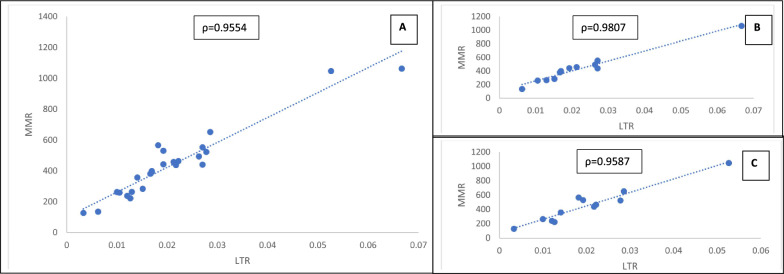
Correlation between Maternal Mortality Ratio (MMR) and Lifetime Risk (LTR) of Maternal Death in Sub-Saharan Africa. **A** Linear relationship between Maternal Mortality Ratio and Lifetime Risk of Maternal Death in Sub-Saharan Africa. **B** Linear relationship between Maternal Mortality Ratio and Lifetime Risk of Maternal Death in Low-income Sub-Saharan Africa. **C** Linear relationship between Maternal Mortality Ratio and Lifetime Risk of Maternal Death in Middle-income Sub-Saharan Africa

Figure 5 presents the linear relationship between LTR-maternal death and MMR. Overall, a strong positive linear relationship similar in direction to that of NID and MMR was seen. Hence a linear rising pattern of association between MMR and LTR with the correlation coefficient of 0.9554. The correlation was slightly stronger in low (rho = 0.9807) than the middle-income (rho = 0.9587) countries though with similar patterns. The linear regression result presented in Table 5 buttress the significant (p < 0.0001) association. The regression shows that a unit increase in LTR-maternal death will lead to about 16,254/100000 MMR in SSA. Also, about 91.3% of the variation in the MMR data was explained by LTR-maternal death. The ID and NID prevalence, difference, MMR and LTR-maternal death are presented in supplementary Table 1.

## Discussion

This study reported the worldwide dominance of African countries in the choice and practice of home birth over health facility-based delivery and the consequential impact on maternal mortality and lifetime risk of maternal death respectively. This was assessed both at the rural and urban divides, and at the low- and middle-income level of the SSA countries, to proffer global strategies that will support program action to sink or reduce the continuous spike in maternal mortality and the related lifetime risk in SSA region.

We observed that NID prevalence is consistently high and above the 10% maximum threshold by WHO to ensure 90% coverage of institutional delivery, and only 3 (South Africa, Rwanda, and Malawi) of the 25 SSA countries studied had achieved this fit by 95.9%, 94.7% and 93.0% health facility delivery coverage respectively. This is evident from higher rate of continuum of care for maternal, newborn and child health in the 3 countries compared to other SSA countries [33]. Our findings similarly reveal that home delivery was more prominent in rural than the urban SSA [34], and the highest prevalent was seen in Chad with about four-fifth followed by Madagascar and Nigeria with approximately three-fifth. We found that one-third of delivery in SSA were in a non-institutional setting. These are in conformance with studies that found the same NID rate in SSA highlighting the high prevalence in Chad and Nigeria [7, 10].

Our study utilized the bivariate and multivariate analysis technique to identify maternal characteristics associated with NID prevalence in SSA and found out that women socio-demographics, obstetrics, and health-related attributes were linked to the NID. Notably were the strength and significance of optimal utilization of ANC and SBA use that decrease the odds of NID by 60% and 98% respectively. The significant effect of optimal ANC and SBA was similarly found to be strongly associated with reduced odds NID by studies in SSA countries including the most affected women population in Nigeria [7, 8, 25].

Furthermore, the odds of NID decrease significantly with increasing levels of women education, wealth, and age. This agrees with the findings that observed that decrease in odds of NID is associated with corresponding decrease in levels of education and socio-economic status among women who had received optimal ANC [8, 25, 35]. However, the odds of NID increase as the levels of women birth order increases and the increase in odds of NID is also associated with increase in countries economic level and thus buttressing the inequality [34]. The increase-increase relationship due to birth order has been described in study investigating predictors of NID among ANC attendees in Nigeria [8]. It’s worth noting that majority of the factors predicting NID in urban areas also predict NID in rural areas of SSA other than the varied country income and sex of household head. This was corroborated by the significant different in urban and rural communities despite the similar NID pattern shown in the comparative study between Ethiopia and Nigeria [27].

The highest positive odd of NID was seen in the African highland nations. This is because the African highland nations were above fifteen times more likely to practice NID than central Africa countries. The odds is approximately nine times higher in east Africa, seven times higher in Southern Africa and six times higher in West Africa. The analysis highlights two specific countries (Chad and Nigeria) as the most affected in West Africa, contributing significantly to the elevated odds observed in the region. Furthermore, the country-level findings reveal about eighty-eight- and thirty-one-times higher odds of NID in these two countries compared to South Africa respectively. Similarly, the odds of NID was approximately thirty-four times higher in Madagascar than South Africa. Also, we found that the rank of odds is consistent in the urban but slightly different in the rural with second highest odds seen in rural Angola while the two most affected west African countries maintained first and third rank in SSA respectively. This further highlights the urban–rural differences [27].

### Implications for maternal health programming and policy

When the impact of NID on MMR was assessed, a positive linear relationship between countries NID and MMR was observed. However, the correlation is stronger in low-income countries with correlation coefficient about twice higher than the middle income countries. The corresponding regression estimate further highlighted the positive strength and direction of association between NID and MMR as a percent increase in home birth will increase maternal mortality ratio by 248 maternal deaths per 100,000 livebirths and thus implies that a rise in home delivery prevalence will contribute to a maternal death per four hundred livebirths in SSA. In other word a maternal death will occur in every four hundred livebirths when home delivery prevalence rise by a percent in SSA. The significant association further shows that non-institutional delivery will explain about one-third of the variation in maternal mortality data in SSA countries. MMR is proportional to the NID prevalence/difference as Chad and Nigeria with highest NID rate are the topmost SSA countries with MMR burden of 1063/100000 and 1047/100000 livebirths respectively [3].

Similarly, the association between NID and the LTR of maternal death showed a direct relationship, with NID increasing as the LTR of maternal death rises and vice versal. The magnitude of association was positively stronger in low-income countries than the middle-income countries. Hence the pattern was apparent in the linear correlation and substantiated by the regression estimate, such that a percent increase in home delivery prevalent is associated with about 8.2/1000 increase in LTR of maternal death in SSA. Implying that the lifetime risk of maternal death rise by 1/122 when home birth increases by 1% in SSA. Also, about 38% of the variation in LTR of maternal death was explained by NID data of SSA countries. Again, highlighting the top two most affected west African countries with the maximum lifetime risk of maternal death of 1/15 and 1/19 among reproductive age women (15–49 years) respectively [3].

Furthermore, the association between women's LTR of maternal death and MMR reveals a strong, positive relationship, showing that the LTR of maternal death rises as MMR increases and vice versal. Hence, the closeness of the correlation coefficient to 1. Though, the relationship strength is slightly higher by 3% in low-income compared to the middle income SSA, the association was substantiated with the significant regression estimates such that a unit increase in lifetime risk of maternal death will result to about 16,254/100000 increase in MMR in SSA. This infer that a maternal death is expected in six livebirths when LTR of maternal death increase by 1 unit across the SSA countries. Hence, LTR-maternal death increase among women of childbearing age as NID magnitude/prevalence and MMR increases and vice versal (3).

Prioritizing institutional deliveries in urban and rural SSA is critical to reducing maternal mortality and achieving the WHO-recommended 90% institutional delivery rate as supported in recent review of antenatal care and skilled birth delivery utilization in SSA [36]. A clear roadmap that addresses infrastructure, human resources, community engagement, and socioeconomic barriers is essential to ensure quality healthcare. However, scaling up healthcare services to reach this goal comes with substantial challenges, including infrastructure limitations, cultural resistance, financial constraints, and weak health systems. Overcoming these challenges will require a coordinated effort among governments, partners, and communities to ensure that maternal healthcare services are accessible, affordable, and used by all. This resonates with the WHO global strategy for human resources for health availability, accessibility, acceptability, quality and effective coverage [37].

### Limitations and strengths of the study

Our study was not entirely free from some drawbacks which are associated with cross-sectional survey/data. Majorly was the possible responder and desirability bias minimized by the survey design with non-response allowance rate as well as probing technique to back-up response. Our study reported snapshot data at a time point and thus limit our conclusions to association only as there is no sufficient information to assess causality. Though we reported countrywide institutional factors indirectly associated with maternal mortality, but our historical data did not measure the clinical (labor and delivery care) predictors such as eclampsia, hemorrhage, etc which are known direct links to maternal mortality. There is potential for minimal type 1 error rate due to multiple statistical tests applied, though was improved by the high data to indicator ratio that strengthen the efficiency of the data for multiple models. However, our study strengths can be observed from the fact that it’s the foremost multicountry observational analysis connecting the non-institutional delivery to maternal mortality outcome in Africa. The use of large, weighted and probabilistic representative data across 25 Sub-Saharan African countries improves the study reliability and accuracy of result herein. Systematic application of statistical technique with guiding principles from univariate to bivariate and to multivariate analysis with descriptive and inferential statistics components added to the scientific rigor. The blend of data from two different sources to produce the holistic results that further strengthen the evidence against the null hypothesis also improves the study precision and lead to reliable conclusion.

### Policy implications and recommendations

Our findings infer that the burden of NID and the connected maternal mortality resides in SSA with interjected country factors explaining the association. A central effort geared towards achieving the WHO recommended 90% uptake of ID in SSA is therefore key to achieve optimal practice. This global strategy should prioritize rural SSA countries and target the most affected countries with negative difference in ID and NID i.e. Chad, Madagascar, Nigeria, and Angola to sink MMR spike. Community advocacy, communication and education program targeting the pregnant women within, and outside ANC should be institutionalized. Governmental and non-governmental organizations should persist in supporting improved access to healthcare and skilled providers particularly in the rural suburbs. Other SSA countries should also learn from what had worked in South Africa, Rwanda, and Malawi particularly the continuum of care strategies if the region is to come close to achieving the SDG target for MMR by 2030. Contextual research on socio-cultural determinant of non-institutional delivery in rural SSA is required to unravel the root-cause.

## Conclusions

One-third of all deliveries in SSA took place outside healthcare facilities with about six in every seven non-institutional deliveries dominant in the rural community. The prevalence of non-institutional birth is highest in Chad, Madagascar, and Nigeria, with Chad and Nigeria among the top three with topmost burden of maternal mortality in SSA. Only South Africa, Rwanda and Malawi had achieved the 90% recommended institutional delivery coverage by WHO. Optimal antenatal care uptake and increased use of skilled birth attendants significantly lower the likelihood of non-institutional births, while higher levels of women’s education, wealth, and age further decrease the chances of home delivery. Notably, compared to South Africa, which has the lowest rates of NID and MMR, the odds of NID are substantially higher in two specific countries, which contributes markedly to the heightened maternal mortality burden observed across West Africa. Though the risk of NID is highest in Africa Highland Nation, NID is positively correlated with both MMR and the LTR of maternal death as a percent increase in NID is associated with a rise in MMR by 248 per 100,000 livebirths and an increase in the LTR of maternal death by 1 in 122.

## Supplementary Information

Additional file 1.

## Data Availability

The anonymize dataset use in this current study is available at the DHS open repository at https://www.dhsprogram.org and the countries DHS report. The generated and analyzed data are available on reasonable request from the corresponding author. Extra data use in the study are available as summary in the supplementary Table 1 and at https://www.who.int/data/gho/data/themes/maternal-and-reproductive-health/maternal-mortality-country-profiles.
