# Supplementary material for: Global dominance of non-institutional delivery and the risky impact on maternal mortality spike in 25 Sub-Saharan African Countries
Source: Glob Health Res Policy. 2025 Feb 27;10:10. doi: 10.1186/s41256-025-00409-x (PMC11866781; doi:10.1186/s41256-025-00409-x)
Supplement: Supplementary file 1 — Additional file 1. [file 41256_2025_409_MOESM1_ESM.pdf]

Supplemental Table1: Country Estimate of Maternal Mortality Ratio, Lifetime Risk of Maternal Death and the Prevalence Difference between Institutional and Non-institutional Delivery in Sub-Saharan Africa

Country	Institutional Delivery Prevalence (%)	Non-institutional Delivery Prevalence (%)	Prevalence difference	MMR/ 100000	LTR/ 1000
Angola	45.7	54.3	-8.6	222	79
Benin	84.6	15.4	69.2	523	36
Burkina Faso	66.2	33.8	32.4	264	77
Burundi	87.9	12.1	75.8	494	38
Cameroon	64.3	35.7	28.6	438	46
Chad	21.4	78.6	-57.2	1063	15
Gambia	84.0	16.0	68.0	458	47
Ghana	72.8	27.2	45.6	263	100
Guinea	51.3	48.7	2.6	553	37
Kenya	60.7	39.3	21.4	530	52
Lesotho	77.7	22.3	55.4	566	55
Liberia	81.2	18.8	62.4	652	35
Madagascar	39.4	60.6	-21.2	392	59
Malawi	93.0	7.0	86.0	381	60
Mali	66.1	33.9	32.2	440	37
Mauritania	68.6	31.4	37.2	464	45
Nigeria	39.6	60.4	-20.8	1047	19
Rwanda	94.7	5.3	89.4	259	95
Sierra Leone	82.8	17.2	65.6	443	52
South Africa	95.9	4.1	91.8	127	300
Tanzania	60.8	39.2	21.6	238	83
Togo	72.2	27.8	44.4	399	59
Uganda	73.9	26.2	47.7	284	66
Zambia	84.2	15.8	68.4	135	160
Zimbabwe	79.5	20.5	59.0	357	71
Average Estimate	66.4	33.6	32.8	439.7	68.9

MMR-Maternal Mortality Ratio; LTR-Lifetime Risk of Maternal Death.

Source: Demographic Health Survey (2014 – 2021) and World Health Organization (2022)

Supplemental Table2: STROBE Statement—checklist of items that should be included in reports of observational studies

	Page No	Recommendation
**Title and abstract**	1-3	(*a*) Indicate the study's design with a commonly used term in the title or the abstract
		(*b*) Provide in the abstract an informative and balanced summary of what was done and what was found
**Introduction**		
Background/rationale	4	Explain the scientific background and rationale for the investigation being reported
Objectives	5	State specific objectives, including any prespecified hypotheses
**Methods**		
Study design	6	Present key elements of study design early in the paper
Setting	6	Describe the setting, locations, and relevant dates, including periods of recruitment, exposure, follow-up, and data collection
Participants	6	(*a*) *Cohort study*—Give the eligibility criteria, and the sources and methods of selection of participants. Describe methods of follow-up<br>*Case-control study*—Give the eligibility criteria, and the sources and methods of case ascertainment and control selection. Give the rationale for the choice of cases and controls<br>*Cross-sectional study*—Give the eligibility criteria, and the sources and methods of selection of participants
		(*b*) *Cohort study*—For matched studies, give matching criteria and number of exposed and unexposed<br>*Case-control study*—For matched studies, give matching criteria and the number of controls per case
Variables	7	Clearly define all outcomes, exposures, predictors, potential confounders, and effect modifiers. Give diagnostic criteria, if applicable
Data sources/ measurement	6-8*	For each variable of interest, give sources of data and details of methods of assessment (measurement). Describe comparability of assessment methods if there is more than one group
Bias	8	Describe any efforts to address potential sources of bias
Study size	6	Explain how the study size was arrived at
Quantitative variables	7-8	Explain how quantitative variables were handled in the analyses. If applicable, describe which groupings were chosen and why
Statistical methods	8-9	(*a*) Describe all statistical methods, including those used to control for confounding
		(*b*) Describe any methods used to examine subgroups and interactions

	(*c*) Explain how missing data were addressed
	(*d*) *Cohort study*—If applicable, explain how loss to follow-up was addressed<br><br>*Case-control study*—If applicable, explain how matching of cases and controls was addressed<br><br>*Cross-sectional study*—If applicable, describe analytical methods taking account of sampling strategy
	(*e*) Describe any sensitivity analyses

Continued on next page

**Results**		
Participants	10*	(a) Report numbers of individuals at each stage of study—eg numbers potentially eligible, examined for eligibility, confirmed eligible, included in the study, completing follow-up, and analysed
		(b) Give reasons for non-participation at each stage
		(c) Consider use of a flow diagram
Descriptive data	10*	(a) Give characteristics of study participants (eg demographic, clinical, social) and information on exposures and potential confounders
		(b) Indicate number of participants with missing data for each variable of interest
		(c) *Cohort study*—Summarise follow-up time (eg, average and total amount)
Outcome data	11*	*Cohort study*—Report numbers of outcome events or summary measures over time
		Case-control study—Report numbers in each exposure category, or summary measures of exposure
		Cross-sectional study—Report numbers of outcome events or summary measures
Main results	10-17	(*a*) Give unadjusted estimates and, if applicable, confounder-adjusted estimates and their precision (eg, 95% confidence interval). Make clear which confounders were adjusted for and why they were included
		(*b*) Report category boundaries when continuous variables were categorized
		(*c*) If relevant, consider translating estimates of relative risk into absolute risk for a meaningful time period
Other analyses	17-18	Report other analyses done—eg analyses of subgroups and interactions, and sensitivity analyses
**Discussion**		
Key results	19-21	Summarise key results with reference to study objectives
Limitations	20	Discuss limitations of the study, taking into account sources of potential bias or imprecision. Discuss both direction and magnitude of any potential bias
Interpretation	19-21	Give a cautious overall interpretation of results considering objectives, limitations, multiplicity of analyses, results from similar studies, and other relevant evidence
Generalisability	22-23	Discuss the generalisability (external validity) of the study results
**Other information**		
Funding	24	Give the source of funding and the role of the funders for the present study and, if applicable, for the original study on which the present article is based

*Give information separately for cases and controls in case-control studies and, if applicable, for exposed and unexposed groups in cohort and cross-sectional studies.

*NA
